# The Influence of Weaving Technologies on the Integral Characteristics of Synthetic Vascular Prostheses

**DOI:** 10.17691/stm2022.14.6.01

**Published:** 2022-11-28

**Authors:** А.А. Shadanov, Т.P. Timchenko, S.V. Vladimirov, P.E. Lushchyk, А.V. Zablotsky, S.О. Kiselyov, I.Yu. Zhuravleva, D.А. Sirota, А.M. Chernyavskiy

**Affiliations:** PhD Student, Research Department of Aorta and Coronary Artery Surgery; E.N. Meshalkin National Research Medical Center of the Russian Ministry of Health, 15 Rechkunovskaya St., Novosibirsk, 630055, Russia;; Junior Researcher, Laboratory of Bioprosthetic Repair, Institute of Experimental Biology and Medicine; E.N. Meshalkin National Research Medical Center of the Russian Ministry of Health, 15 Rechkunovskaya St., Novosibirsk, 630055, Russia;; Engineer, Laboratory of Bioprosthetic Repair, Institute of Experimental Biology and Medicine; E.N. Meshalkin National Research Medical Center of the Russian Ministry of Health, 15 Rechkunovskaya St., Novosibirsk, 630055, Russia;; Deputy General Director for Innovation Activity; Science and Technology Park of the BNTU “Polytechnic”, 37/1 Surganova St., Minsk, 220013, Republic of Belarus; Chief of Innovative Production Center for Medical Devices; Science and Technology Park of the BNTU “Polytechnic”, 37/1 Surganova St., Minsk, 220013, Republic of Belarus; Production Engineer; Science and Technology Park of the BNTU “Polytechnic”, 37/1 Surganova St., Minsk, 220013, Republic of Belarus; Professor, Director of the Institute of Experimental Biology and Medicine; E.N. Meshalkin National Research Medical Center of the Russian Ministry of Health, 15 Rechkunovskaya St., Novosibirsk, 630055, Russia;; Head of the Research Department of Surgery of Aorta, Coronary and Peripheral Arteries, Institute of Blood Circulation Pathology; E.N. Meshalkin National Research Medical Center of the Russian Ministry of Health, 15 Rechkunovskaya St., Novosibirsk, 630055, Russia;; Professor, Corresponding Member of the Russian Academy of Sciences, General Director; E.N. Meshalkin National Research Medical Center of the Russian Ministry of Health, 15 Rechkunovskaya St., Novosibirsk, 630055, Russia;

**Keywords:** vascular prosthesis, woven dacron prostheses, water permeability, kinking radius

## Abstract

**The aim of the study** is to determine physical and structural properties of woven synthetic prostheses depending on the type of the weave.

**Materials and Methods:**

Ten vascular prostheses manufactured at the Science and Technology Park of the BNTU “Polytechnic” (Minsk, Republic of Belarus) have been analyzed. The prostheses differed in the type of weaving, duration and temperature of thermal fixation during crimping. Three samples had a single-layer structure and 7 samples had a double-layer structure. Tests for water permeability, resistance to radial bending, and porosity of the prostheses have been performed.

**Results:**

The single-layer woven prostheses have demonstrated a low level of water permeability: the best result was shown by sample No.1: 80 [77.1; 80.5] ml/min/cm^2^. A strong direct correlation was revealed for these prostheses: the larger the pore diameter, the greater permeability (r=0.778; p=0.05). The single-layer woven prostheses appeared to be most resistant to radial bending, samples No.1 and 3 had no deformations at the minimum radius of the cylinder (r<4 mm), sample No.2 showed deformation on the cylinder with r=5 mm. For the single-layer prostheses, a strong negative correlation was noted (r=‒0.97; p=0.04) between the density of the warp threads and the kinking radius.

All double-layer prostheses have demonstrated higher water permeability and weak resistance to deformation during radial bending. Samples No.4 and 8 were found to have minimum and maximum water permeability of 276.5 [258.3; 288.4] and 538.8 [533.3; 564.3] ml/min/cm^2^, respectively. The minimum kinking radius (7 mm) was shown by samples No.9 and 10. The worst results were demonstrated by sample No.6, which was deformed with minimal bending.

**Conclusion:**

Samples with ordinary plain weave have a low level of water permeability and high resistance to radial deformation, which makes them look most promising for the application in vascular surgery.

## Introduction

At the dawn of vascular surgery, various nonporous hollow tubes (from vitallium, rigid plastic, etc.) were used at the first attempts to replace pathologically changes arteries. As the procedure was often accompanied by early thromboses or occlusions [1, 2], these prostheses were soon abandoned. A high risk of complications is associated with the absence of channels or space in the wall of the nonporous tubes for cellular filling providing formation of neointima on the luminal surface, which is of critical value for a long-term patency of a vascular implant [3-5].

In the second half of the XX century, textile-based vascular prostheses have been proved to be optimal for the replacement of large-diameter arteries [6, 7], since they were capable of vascular wall neovascularization and formation of functional neointima owing to their porosity. This has a favorable effect on a long-term patency of a vascular prostheses [5, 8].

In 2018, 130 thousand interventions on aorta and peripheral arteries were performed in Russia; the annual increase amounted to 8.4% [9, 10]. There are no exact data in the open access on the application of synthetic vascular prostheses in Russia and in the world. However, only the demand in the prostheses for the reconstruction of thoracic and thoracoabdominal parts of aorta may be estimated at about 50,000 [11] in the world and 800 in Russia [10].

**Figure 1.**
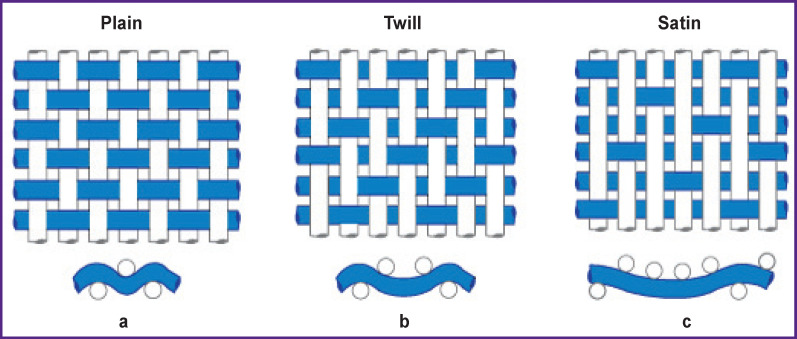
Rapports of the main types of textile weaves: (а) Rwa/Rwe 2/2 plain weave; (b) 2/1 warp twill weave; (c) 4/1 satin weave. The fibers of the warp threads are located vertically, the fibers of the weft threads are horizontal (pattern from site https://www.nicepng.com/maxp/u2w7r5e6i1t4e6e6/)

The most popular vascular prostheses are fabricated from nonwoven polytetrafluorethylene (PTFE, teflon) or from the fibers of polyethylene terephthalate (PET, lavsan, dacron) [12]. Prostheses from polyester fiber are knitted or woven vessels with various weaving and knitting rapports influencing graft properties in different ways [13]. A woven prosthesis consists of two interlaced, perpendicularly located systems of warp and weft polyester fibers. Vertical fibers correspond to the warp, horizontal ones to the weft (Figure 1), providing a dense and strong structure with a relatively low porosity. The number of threads forming a complete pattern is called a weaving rapport.

There are rapports of the warp (Rwa) determined by the number of the warp threads forming it, and of the weft (Rwe) determined by the number of the weft threads. Rapports of the main types of interweaving: plain, twill, and satin are presented in Figure 1.

Knitted prostheses are fabricated on the knitting machines using the warp or flat knitting technique [12]. The distance between the threads and fabric porosity depend on the needle size and thread diameter. Knitted prostheses possess higher porosity, softness, flexibility, and stretchability as compared to the woven prostheses, which is an advantage, on the one hand, while, on the other, often results in intraoperative bleeding, dilatation, and graft aneurisms [11]. Both woven and knitted prostheses are further subject to crimping to improve stretchability, flexibility and to preserve the internal lumen if the kinking radius increases.

The main requirements to the vascular prostheses are determined by ISO 7198:2016 including also the requirements to mechanical characteristics, biocompatibility, biostability, and biofunctionality [14]. Theoretically, the density and the type of the weaving rapport must influence permeability of the prosthetic graft wall and its mechanical properties: strength, flexibility, hemocompatibility (due to the luminal surface relief), and the intensity of transmural neovascularization. However, this question is not reflected in the literature in the consistent manner: only scattered data about separate characteristics of some commercial vascular grafts are encountered.

**The aim of our investigation** was to study the effect of various weaving rapports and crimping modes on the microstructure, kinking radius, and permeability of woven vascular prostheses.

## Materials and Methods

Ten corrugated vascular prostheses fabricated from 74dtex S110 polyethylene terephthalate fibers (Gruschwitz Textilwerke AG, Germany) at the Science and Technology Park of the BNTU “Polytechnic” (Minsk, Republic of Belarus) were used in our work. These prostheses differed in the weaving technique, temperature and duration of thermal fixation (Table 1).

**Table 1:** Technological characteristics of vascular prostheses

Sample No.	Prosththesis diameter (mm)	Density*		Number of layers	Thermal fixation during crimping	
		Rwe	Rwa		Duration (min)	t (°C)
1	20	87	116	1	10+10^х^	210
2	8	65	122	1	10	210
3	10	94	94	1	10	210
4	12	95	129	2	10	200
5	12	67.5	95	2	13	170
6	12	45	89	2	13	170
7	10	60	93	2	13	170
8	12	45	89	2	10	190
9	10	60	93	2	12	180
10	8	55	110	2	10	190

* number of fibers per 1 cm of warp (Рwa) and of weft (Pwe); ^х^ in the process of crimping, the prosthesis underwent repeated thermal fixation of 10 min duration at 210°С.

The microstructure of each prosthesis has been studied and tests for permeability and resistance to radial bending have been performed.

### Evaluation of the prosthesis microstructure using scanning electron microscopy

Two 3×3 mm samples were prepared from each preliminarily disinfected and dedusted prosthesis. Each sample was fixed to the microscope stage with its internal or external surface using a carbon double-sided adhesive tape. After fixation, the sample surface was coated with a 20-nm carbon layer in argon atmosphere at 6-mA ion current and pressure of 0.1 mm Hg using Q150T ES system (Quorum Technologies, Great Britain) and examined with the help of MIRA 3 scanning electron microscope (Tescan, Czech Republic). The obtained images were analyzed in the ImageJ program to acquire qualitative data on the weaving structure and quantitative data on pore dimensions.

### Evaluation of water permeability

**Figure 2.**
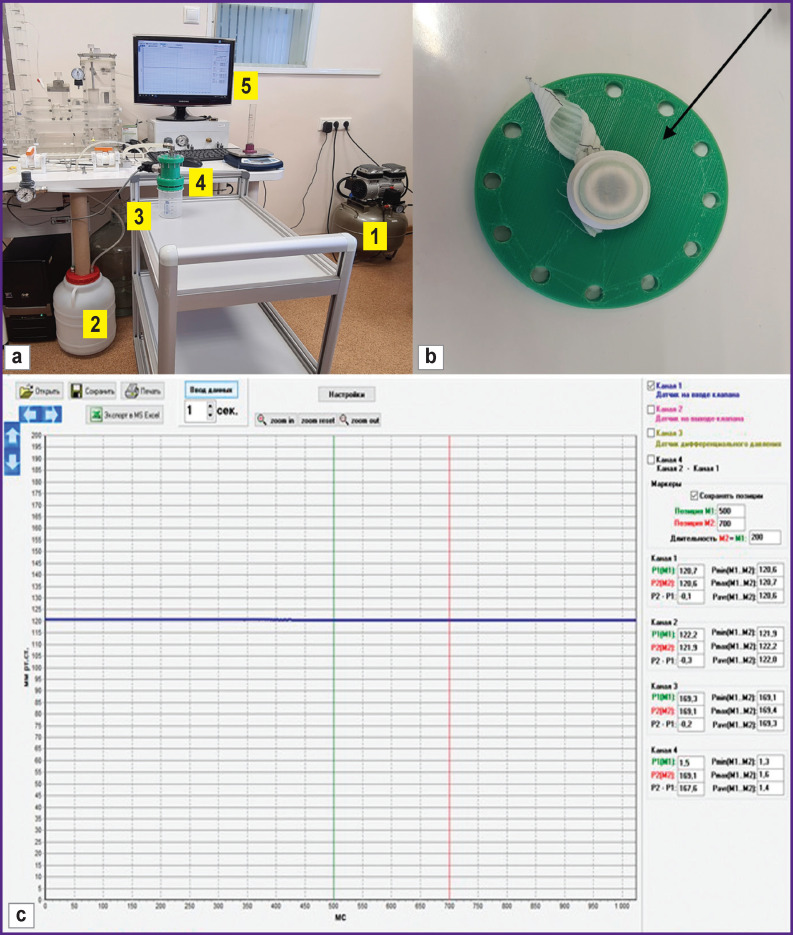
Water permeability testing device: (а) test bench; general view: *1* — air compressor; *2* — distilled water tank; *3* — BD Sensors DMP 331 water pressure sensor, mounted into the holding device; *4* — sample holding device; *5* — monitor for controlling liquid pressure in the system; (b) fixation of the sample in the straightened state with a rubber ring (*black arrow*) into the 0.78-cm^2^ aperture; (c) continuous monitoring and display of pressure values in the system during testing procedures

Permeability was determined by the velocity of distilled water flow through the prosthesis wall. The tests were conducted at room temperature in the original system (Figure 2 (a), (b)), designed in compliance with GOST R ISO 7198—2013.

The test bench was composed of:

1. a device for determining permeability including BD Sensors DMP 331 water pressure sensor (BD SENSORS RUS, Russia) sensible in the range of 0–0.4 bar, and a sample holding system (see Figure 2 (a), (b));
2. a unit for bench pressure control to check hydrodynamic characteristics of heart valve prostheses with a software (NPP MedEng, Russia);
3. oil-free air compressor, distilled water tank (10 L), connecting pipes and valves, measuring container (see Figure 2 (а)).

After maximal stretching of the crimped folds, each examined sample was hermetically fixed in the system with rubber seals (see Figure 2 (b)); the cross-sectional area of the holding system aperture was 0.78 cm2. The hydrostatic pressure in the system was gradually increased up to a constant level of 120±2 mm Hg and controlled on the bench monitor (Figure 2 (c)). The volume of liquid passing through the prosthesis wall per 1 minute was thereafter fixed by means of the measuring container. The test was repeated 5 times for each sample. After the completion of the tests, the rate of water outflow per 1 cm^2^ was calculated by the formula:

Water permeability=QA,

where *Q* is the flow rate through the sample (ml/min), *A* is the cross-sectional area of the holding system aperture (cm^2^).

### Tests for resistance to radial bending

**Figure 3.**
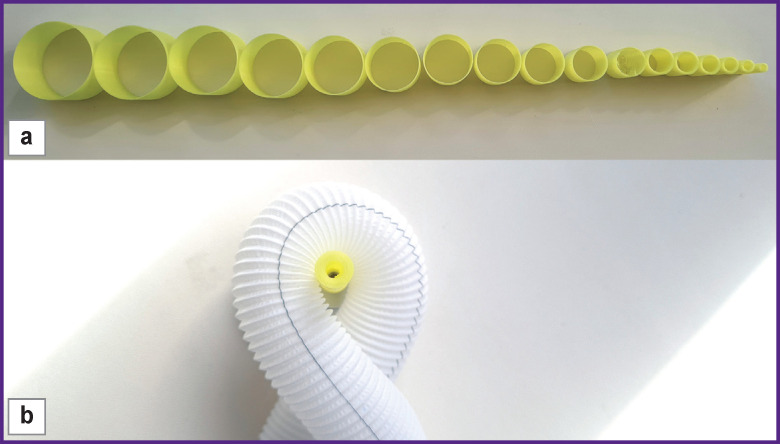
Tests for resistance to radial bending: (а) cylindrical gauges with the 4.0‒42.5-mm radius and 1.5-mm pitch; (b) loop formation for sample No.1, cylindrical gauge diameter — 8 mm (R=4 mm)

Kink radius was measured using a cylinder gauges of a specific diameter with the radius in the range of 4.0–42.5 mm and a 1.5-mm pitch (Figure 3 (а)).

A loop was formed from the tested samples, the cylinder gauge with maximum diameter was placed inside of it, the ends of the prosthesis were tightened in opposite directions to reduce the loop (Figure 3 (b)) with subsequent replacement by the gauge of a smaller diameter. The test was repeated until a kink or deformation reducing the prosthetic lumen appeared. The gauge size giving a kink/deformation of the prosthesis was considered the value of the bend or kink radius.

### Statistical processing of the results

Results were statistically processed using the Stata 14.0 program. The Shapiro–Wilk test was applied to check normality of distribution in each group. Taking into account a small sample volume, the quantitative data were presented as median and interquartile range (25^th^ and 75^th^ percentiles). Statistical significance of differences was determined using the Mann–Whitney U test, considering the differences statistically significant at р<0.05. The correlation analysis was employed using Pearson correlation coefficient (r), the Chaddock scale was used for result interpretation.

## Results

### The structure of prosthetic tissue

**Figure 4.**
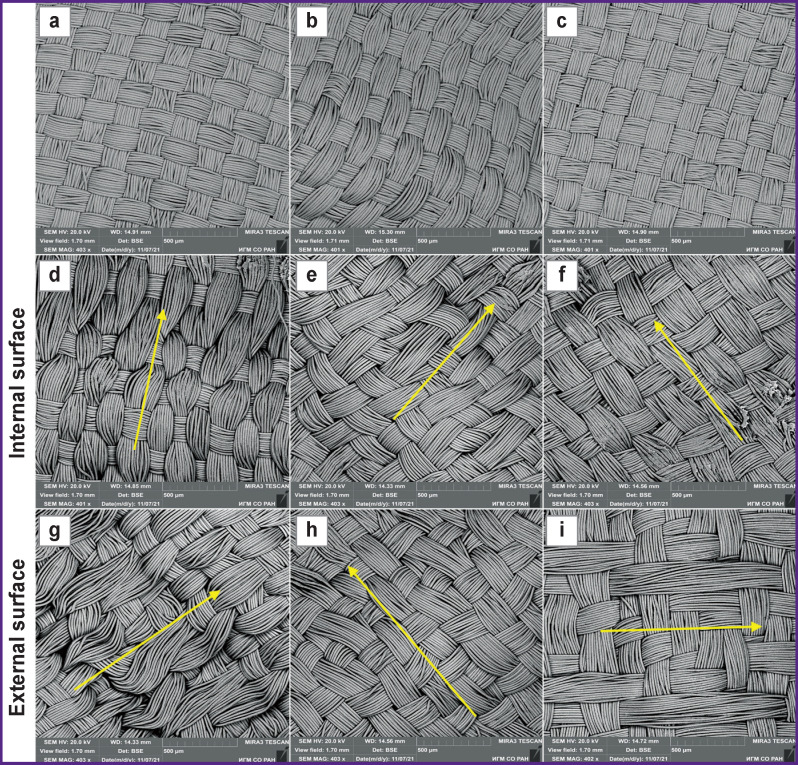
Prostheses samples: (а) prosthesis No.1; (b) prosthesis No.2; (c) prosthesis No.3; (d) and (g) prosthesis No.4; (e) and (h) prosthesis No.5; (f) and (i) prosthesis No.6. Arrows show the direction of the warp threads

Prosthetic samples No.1–3 (Figure 4 (a)–(c)) had a plain weave where the rapport of the weft and warp is 2 to 2 (Rwe=Rwa=2).

Samples No.4–10 are characterized by a complicated two-layer weave. The internal surface of sample No.4 has a plain weave with the rapport of the weft and warp presented as 2 to 2 (Rwe/Rwa=2/2). The external surface is a 5/3 shaded satin, there is also bifurcation of the warp threads with weft wrapping (Figure 4 (d), (g)). Sample No.5 is presented by a complicated weave based on the plain weave, but on the internal and external prosthetic surface there are bifurcations of the warp threads with the wrapping of the weft yarns (Figure 4 (e), (h)). The internal surface of sample No.6 has a plain weave characterized by bifurcation of the warp thread fibers. On the external surface, there are signs of 2/1 twill weave with a positive diagonal line located at the angle of 45°, which is directed from the bottom upwards and from left to right (plain twill) and is formed by the warp threads. Additionally, there is also 6/4 satin weave (Figure 4 (f), (i)).

**Figure 5.**
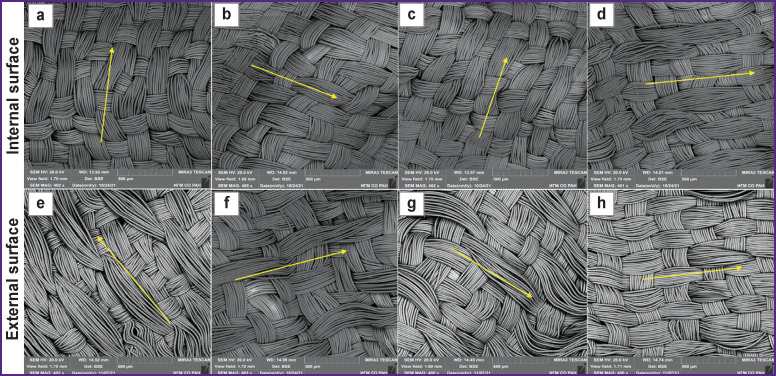
Prostheses samples: (а) and (e) prosthesis No.7; (b) and (f) prosthesis No.8; (c) and (g) prosthesis No.9; (d) and (h) prothesis No.10. Arrows show the direction of the warp threads

The internal surface of prosthesis No.7 had a 2/2 rep weave of the warp (derivative of a plain weave), with the separation of the warp thread fibers and wrapping of the welt thread. The external surface is represented by a complicated weave combining the ordinary plain weave with the warp rep weave. The satin weave was also additionally noted. In some areas, the warp threads were separated and embraced the threads of the warp and weft (Figure 5 (a), (e)). The internal surface of sample No.8 had a complicated twill weave characterized by the separation of the weft threads and their passage through the warp threads, “splitting” the latter. The external surface had a twill weave in the form of a negative diagonal line located at the angle of 45° and directed from the bottom upwards and from right to left (2/1 inversely shifted twill) and formed by the warp threads. Supplementary, satin weave 6/4 was also observed (Figure 5 (b), (f)). Prosthesis No.9 was represented by a complicated double-faced weave with two systems of weft and warp. On the internal surface of the prosthesis, there was a 2/2 plain rep weave, and two warp threads bifurcating with wrapping of the weft threads. On the external surface, a plain weave was combined with a plain rep and a supplementary 5/3 satin weave (Figure 5 (c), (g)). The internal surface of sample No.10 had a plain weave of the warp. Additionally, 5/4 satin weave was noted. The external surface had a plain weave of the warp with bifurcation of the warp threads (Figure 5 (d), (h)).

The results of pore measurements are presented in Table 2.

**Table 2:** Technical characteristics of the examined vascular prostheses, Me [Q1; Q3]

Sample No.	Pore diameter (μm)		р	Water permeability (ml/min/cm^2^)	Kinking radius, R* (mm)
	Internal surface	External surface			
1	14.7 [13.1; 17.6]	14.2 [11.6; 16.6]	0.42	80.0 [77.1; 80.5]	<4
2	22.0 [15.6; 25.1]	15.9 [14.6; 17.9]	0.08	382.4 [382.0; 383.3]	5
3	19.0 [18.6; 21.8]	18.4 [14.7; 20.6]	0.19	145.3 [135.9; 156.9]	<4
4	29.4 [21.2; 39.8]	24.7 [19.9; 29.4]	0.44	276.5 [258.3; 288.4]	17.5
5	25.8 [21.8; 32.0]	25.1 [17.3; 33.7]	0.87	373.0 [355.1; 392.5]	15
6	29.4 [25.8; 31.9]	24.3 [21.2; 30.0]	0.12	312.1 [296.1; 319.2]	>42.5
7	45.4 [33.5; 59.2]	32.8 [24.3; 39.8]	0.06	397.4 [372.4; 428.2]	21
8	31.5 [22.4; 42.5]	33.1 [32.1; 50.7]	0.22	538.8 [533.3; 564.3]	12.5
9	30.9 [25.4; 35.7]	25.2 [22.2; 30.8]	0.17	493.5 [472.4; 504.5]	7
10	43.1 [27.9; 50.6]	33.1 [29.1; 38.7]	0.38	338.4 [316.6; 373.0]	7

* R corresponds to the cylinder diameter causing deformation of the prosthesis: R<4 mm— absence of deformation or crease on the minimum cylinder gauge; R>42.5 mm — corresponds to the maximum cylinder gauge causing deformation of the prosthesis.

### Permeability of the examined prostheses

The results of water permeability tests are also given in Table 2. The least permeability of 80 [77.1; 80.5] ml/min/cm^2^ was shown by single-layer sample No.1 with the minimum pore diameter. Besides, a strong direct correlation was found for the single-layer prostheses: the larger the pore diameter, the greater permeability (r=0.778; р=0.05). This relation was not detected for the double-layer prostheses (r=0.04; р=0.01). A strong negative correlation was observed between the fluid volume passing through the prosthetic wall and the density of the weaving rapport (the total density of the warp and weft threads) both for the single-layer (r=–0.708; р=0.05) and double-layer prostheses (r=–0.577; р=0.05).

On the whole, permeability of the double-layer prostheses was higher than that of single-layer. At the same time, the differences within this group do not have consistent patterns. Thus, the highest permeability was demonstrated by double-layer prostheses No.8 and 9, although the pore sizes on their internal and external surface (25.2–33.1 μm) were smaller than in samples No.7 and 10 (33.1–45.4 μm). Permeability of samples No.8 and 9 was higher than in samples No.6 and 7 despite the fact that the total thread density of the weft and warp was identical in No.6 and 8, and No.7 and 9.

### Resistance to radial bending

The results of the tests for resistance to radial bending (see Table 2) have shown that the kinking radius does not depend on the diameter of the tested prosthesis (r=0.0036; р=0.01). Samples No.1 and 3 had the greatest resistance to bending (R<4), and a strong negative correlation (r=–0.97; р=0.04) was noted between the density of the warp threads and the bend radius. The kinking radius of the double-layer prostheses was much larger while the negative correlation between the thread density and the kinking radius was much weaker (r=–0.238; р=0.01). The worst results were demonstrated by double-layer prosthesis No.6 which was deformed at minimal bends, while the best results were shown by prostheses No.9 and 10 with the kinking radius of 7 mm. Besides, no statistically significant relations have been found between the kinking radius and thermal fixation modes during crimping.

## Discussion

For several decades, dacron (polyethylene terephthalate) has remained the main material from which vascular prostheses were fabricated for the large-diameter arteries (8 mm and larger). In the modern medical market, 95% of vascular prostheses are made of polyethylene terephthalate, woven or knitted, and only 5% are presented by more modern materials: nonwoven polytetrafluorethylene or polyurethane [13, 15, 16]. The discussion is going on among surgeons as to what prostheses preference should be given: woven or knitted. Knitted prostheses are more stretchable, flexible, and porous. The latter property facilitates neovascularization of the wall, on the one hand, but increases blood permeability, on the other, contributing to intra- and perioperative bleedings which predetermines obligatory use of a sealing coating [13, 17]. Woven prostheses make it possible in the process of intraoperative modeling and suturing to obtain a smoother cut-off edge without fiber and stitch stretching. Besides, they are stronger and less permeable for blood [18].

Striving to obtain a more hermetic wall, one should remember that pores not less than 25 μm are necessary for the appropriate integration of a prosthesis [19, 20], since the diameter of the functioning arteriole (endothelium and one layer of smooth muscle cells) is on average 23.1±13.1 μm [5], since in neovascularization, the growing endothelial cells are followed by the smooth muscle ones [21, 22].

It has been previously believed that plain weave limits neovascularization and neointima formation [23]. Sateen weaves, increasing porosity and permeability of prostheses, were introduced as an alternative [24-26]. However, later it was proved that satin weave does not ensure complete wall vascularization of the vascular prostheses [27].

When studying prostheses with a plain weave which differ in linear density and the number of fibers in the threads of the warp and weft, Mokhtar et al. [28] have proved that porosity of the prosthesis does not correlate with permeability. High water permeability is believed by the authors to be associated with thin fibers in the warp threads, which increases the space between the threads and contributes to leakage. The authors came to the conclusion that the most important parameter for vascular prostheses with the plain weave is the fabric density depending on the linear density of the yarn and threads.

In our study, positive correlation was found between the increase of the pore diameter and the level of water permeability for the single-layer prostheses (r=0.778; р=0.05), but not for the double-layer ones (r=0.04; р=0.01). The greatest permeability was demonstrated by double-layer prostheses No.8 and 9, although the pore sizes on their internal and external surfaces were smaller than in samples No.7 and 10.

There is no consensus about the effect of thread density on permeability of woven prostheses. For example, Guidoin et al. [29], Pourdeyhimi and Text [30] have shown in their investigations that permeability grows with the increase of the thread number in the bundles of the textile weave. According to the data obtained by Mokhtar et al. [28], the increase of the thread number in the plain weave reduced prosthesis permeability. We believe that such unambiguous relation may be sought only for single-layer prostheses (not for double-layer) where a general rapport of the cloth has a significant effect on permeability. Thus, permeability of samples No.8 and 9 was higher than that of No.6 and 7 in spite of the fact that the total density of the weft and warp threads was identical in No.6 and 8, and No.7 and 9, respectively. It is likely that in complicated types of the weave, “looseness” of the general rapport is of great importance, whereas in the simple plain weave, the increase of the thread number in the warp or weft reduces permeability of prostheses due to greater cloth density.

Guan et al. [31] have analyzed vascular prosthesis permeability for water, plasma, and whole human blood. Woven grafts with a plain weave (monofilament in the warp and polyfilament in the weft) differing in the number of weft threads were investigated. The weft density has been established to correlate negatively with porosity and water permeability but positively with the strength of the prostheses. However, blood permeability did not depend much on the weave density.

As a rule, woven synthetic prostheses undergo crimping in order to impart cylindrical framing, stretchability, and resistance to bending in the zones of natural anatomical curves (thoracic part of aorta, projection of inguinal fold, and so on). Resistance to bending determines the patency of the vascular graft in the early period after implantation. In this connection, the kinking radius, according to the GOST R ISO 7198—2016, is one of the main criteria of prosthesis evaluation [14]. The single-layer prostheses have been established to possess significantly greater resistance to radial bending than double-layer. And the greater the density of the warp threads, the smaller the kinking radius. Such dependence has not been detected for the double-layer prostheses. Besides, it has been shown by us that the kinking radius does not depend on the diameter of the examined prosthesis (r=0.0036; р=0.01), which will allow one to compare this parameter obtained in the process of testing prostheses of various diameters.

A crimping procedure is accompanied by a thermal processing to fix the shape, which theoretically may affect the kinking radius and permeability. However, despite a large number of works on the evaluation of mechanical properties of prostheses, there are no data in the literature about resistance to bending depending on the crimping method and the types of weaving of the factory-made prostheses [12, 16, 32]. Therefore, it should be noted that in our study we have not found reliable relations between the kinking radius and modes of thermal fixation during crimping.

Thus, based on the complex of the studied characteristics, single-layer prostheses with the plain weave and warp/weft density of 87/116 threads/cm^2^, the diameter of not less than 20 μm, water permeability in the range of 75–80 ml/min/cm^2^, and the kinking radius not exceeding 4 mm (sample No.1) are considered by us most promising. It is these prostheses that will be used in further investigations when developing sealing agents with antibacterial properties.

It should be taken into consideration that in our study samples were fabricated from the same fiber. It is clear that the material and thickness of the fiber can also influence biotechnical characteristics of woven prostheses. In this connection, these variable parameters should be taken into account when conducting comparative tests of the prostheses fabricated by various manufacturers or from different fibers.

## Conclusion

The type of weave and general rapport of woven vascular prostheses influence significantly their biotechnical properties. Prostheses with a double-layer weave are characterized by increased water permeability and are less resistant to radial bending. On the whole, single-layer prostheses have low water permeability and a small kinking radius. Of the 10 examined prostheses, made from polyethylene terephthalate fiber, the single-layer prosthesis with a plain weave and warp/ weft density of 87/116 threads/cm^2^ possesses optimal flexibility and water permeability.
